# Continuous glucose monitoring evidence of celiac disease in type 1 diabetes

**DOI:** 10.1515/jpem-2025-0302

**Published:** 2025-09-25

**Authors:** Jessica L. Ruiz, Lisa A. Asaro, Allison S. Bernique, Elizabeth Healey, Jocelyn A. Silvester, David Wypij, Michael S.D. Agus, Christina M. Astley

**Affiliations:** Department of Pediatrics, 1862 Division of Endocrinology Boston Children’s Hospital, Boston, MA, USA; Department of Pediatrics, Harvard Medical School, Boston, MA, USA; Department of Cardiology, Boston Children’s Hospital, Boston, MA, USA; Program in Health Sciences and Technology, Massachusetts Institute of Technology, Cambridge, MA, USA; Computational Health Informatics Program, Boston Children’s Hospital, Boston, MA, USA; Department of Pediatrics, Division of Gastroenterology, Hepatology and Nutrition, Boston Children’s Hospital, Boston, MA, USA; Department of Biostatistics, Harvard T.H. Chan School of Public Health, Boston, MA, USA; Department of Pediatrics, Division of Medical Critical Care, Boston Children’s Hospital, Boston, MA, USA; Computational Epidemiology Lab, Boston Children’s Hospital, Boston, MA, USA; Broad Institute of MIT and Harvard, Cambridge, MA, USA

**Keywords:** continuous glucose monitoring, celiac disease, pediatric diabetes, postprandial, type 1 diabetes

## Abstract

**Objectives:**

Quantitative glycemic metrics are needed to identify undiagnosed celiac disease in type 1 diabetes and reduce delays in celiac diagnosis. Celiac enteropathy drives malabsorption that increases the risk of prandial insulin-glucose mismatch and hypoglycemia. We assessed if children with type 1 diabetes and celiac disease have lower post-prandial glucose levels preceding celiac diagnosis vs. those without celiac disease, leveraging continuous glucose monitoring (CGM) data and a computational meal annotation algorithm.

**Methods:**

In this retrospective cohort study, children with type 1 diabetes <12 months duration using CGM, positive celiac serologies and biopsy confirmed celiac disease (n=16) were matched 1-to-4 to those with negative celiac serologies (n=60). Meals were computationally annotated in the 30-day window before serologies. Differences in post-prandial trough glucose and other prandial glycemic outcomes were assessed via mixed models.

**Results:**

Undiagnosed celiac disease was associated with a lower glucose rise from meal start to peak vs. no celiac disease (−8.9 %, 95 % CI, −14.9–−2.5 %, p=0.009) and, during the first meal of the day, a lower fall from peak to trough (−9.3 %, 95 % CI, −16.5 %–−1.5 %, p=0.02). There was no significant association between celiac disease and trough glucose, meal hypoglycemia or time hypoglycemic.

**Conclusions:**

Computational analysis revealed that blunted prandial glycemic trajectories, not hypoglycemia, are associated with undiagnosed celiac disease in children with type 1 diabetes using CGM. These findings challenge current guidelines, and future studies should validate and integrate these glycemic biomarkers into a CGM-based model for real-time prediction of celiac disease in type 1 diabetes.

## Introduction

Timely diagnosis of celiac disease (CD) among children with type 1 diabetes mellitus (T1D) is imperative to mitigate complications, yet diagnosis remains challenging. Patients with T1D have a higher prevalence of CD [1], [2], [3], [4], [5] (1–16.4 % [4] in T1D vs. 0.2–5.5 % [6] overall) but people with T1D are more often asymptomatic [1], [7], [8], [9] and classic CD symptoms are unreliable predictors of the disease in this population [10]. Treatment of CD is crucial to prevent lower bone mineral density [11], poor growth and gastrointestinal malignancy [4], as well as complications with the dual CD and T1D diagnosis, including an increased risk of dyslipidemia [12], 13] and microvascular disease [14], 15].

Enteropathy secondary to CD impairs intestinal digestion and nutrient absorption [16]. In people with T1D, carbohydrate malabsorption increases the risk of mismatch between prandial insulin and serum glucose, which can alter post-prandial glucose patterns including an increased risk of hypoglycemia. While increased or unexplained hypoglycemia is a guideline-based indication to screen for CD in patients with T1D [4], 5], clinicians lack guidance on quantitative metrics of hypoglycemia or other continuous glucose monitor (CGM) data features that might indicate CD. Past pediatric T1D studies, predating common CGM use, demonstrated higher rates of hypoglycemia [17], 18] and/or lower hemoglobin A_1c_ (HbA_1c_) [19] at CD diagnosis, though subsequent studies have not replicated these findings [8], 20], 21].

CGM measure high-resolution glucose data, providing novel, granular insights into glucose dynamics, namely, the magnitude and pace of glucose rise and fall. Computational analyses of CGM prandial glucose dynamics are an emerging tool to distinguish glycemic features of underlying pathology [22], and may identify a signature of insulin-glucose mismatch and dysglycemia associated with CD to help guide clinical care. Toward this end, we tested the hypothesis that exposure to malabsorption from CD in T1D increases post-prandial hypoglycemia and alters prandial glycemia in a retrospective cohort of children with new-onset T1D using CGM prior to CD diagnosis.

## Materials and methods

### Study cohort

We performed a retrospective analysis of a matched cohort drawn from a study cohort of children aged 2–21 years followed in the Boston Children’s Hospital (BCH) T1D program. This study was conducted in accordance with the Declaration of Helsinki. This study protocol was reviewed and received Ethical Approval from the Boston Children’s Hospital Institutional Review Board, approval number IRB-P00032591 on July 12, 2019. The need for written informed consent was waived for this retrospective study by the Institutional Review Board.

The study cohort included individuals diagnosed with T1D from January 1, 2015 (beginning of BCH CGM database) to December 30, 2022. Eligible subjects did not have a diagnosis of CD at the time of diabetes diagnosis, underwent celiac serologies <12 months following diabetes diagnosis, used Dexcom CGM (DexCom, Inc., San Diego, CA, USA) in the month prior to serologies and shared CGM data with BCH via the cloud-based Dexcom Clarity Portal. We excluded children using automated insulin delivery technology including predictive low glucose suspend features and/or with gastrointestinal comorbidities (e.g. inflammatory bowel disease).

### Exposure definition

**Figure 1:**
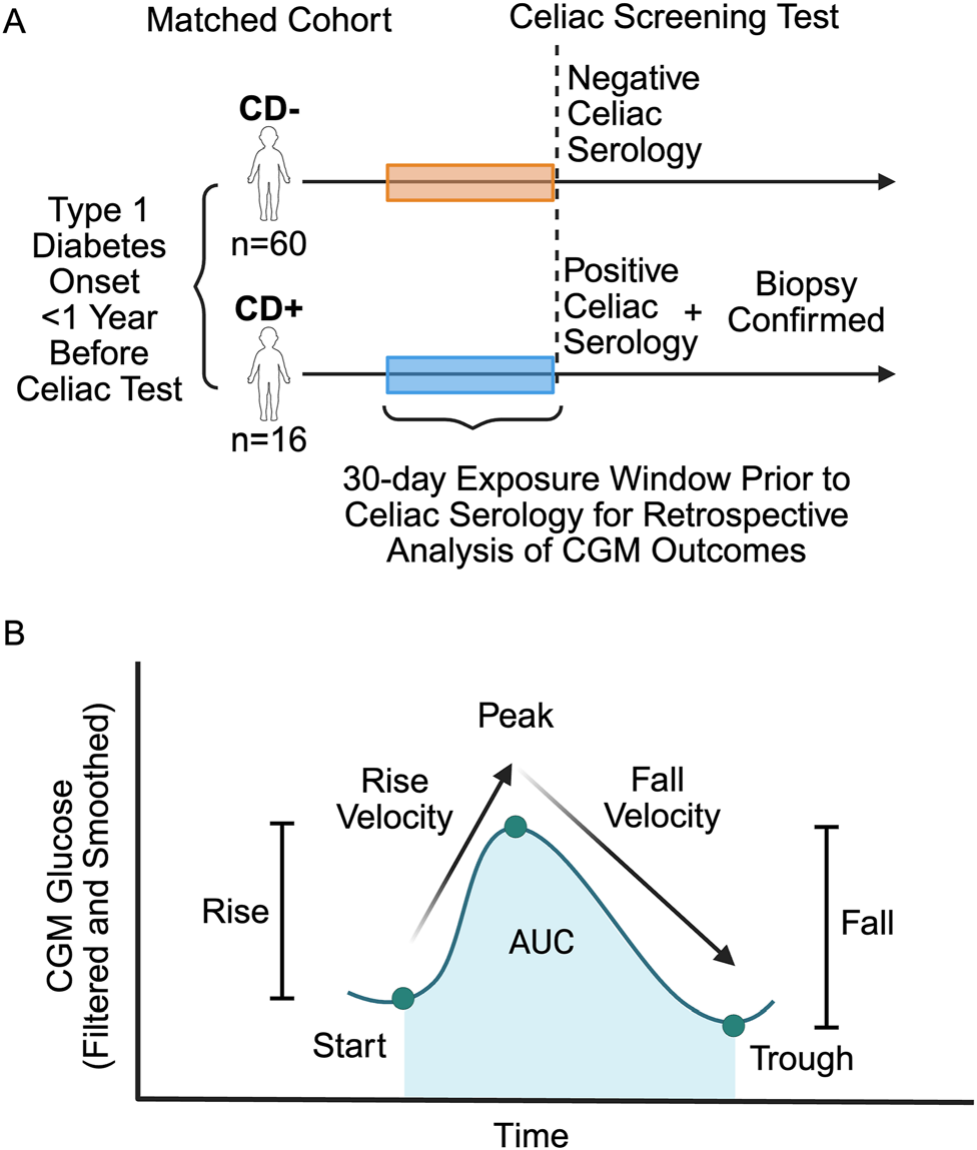
Study design and prandial outcome definitions. (A) Schematic of cohort characteristics and timing of celiac screening test (measurement of exposure) relative to the window for CGM outcome analysis in the matched cohort. (B) Schematic depicting definitions of prandial glucose outcomes. Meals were inferred by first identifying a glucose peak with a subsequent glucose trough. The meal glucose start was defined as an inflection from downtrending to uptrending glucose preceding the peak. Meal glucose rise (rise) was calculated as peak minus start and rise velocity was calculated as rise divided by time from start to peak. Meal glucose fall (fall) was calculated as the absolute value of trough minus peak and fall velocity was calculated as fall divided by time from peak to trough. CD−, no celiac disease; CD+, undiagnosed celiac disease; CGM, continuous glucose monitoring; AUC, area under curve. Created in BioRender. Ruiz, J. (2025) https://BioRender.com/2x4rz0m.

The exposure of interest was undiagnosed CD. The exposure window was the 30 days prior to a documented celiac antibody testing date (i.e., index date) (Figure 1A). The index date for one individual was the date of follow-up serologies after initial positive serologies, as they started CGM in the interim but were advised to continue consuming gluten with documented gluten intake between serologies. Confirmed CD (CD+) was defined using the gold standard, i.e., duodenal histologic findings of crypt hyperplasia, villous atrophy and intraepithelial lymphocytosis plus elevated tissue transglutaminase IgA [23]. Those unexposed to CD (CD−) were defined as those with normal CD serologies.

### Matched cohort criteria

Demographic and clinical data were collected via retrospective chart review. Potential subjects were only eligible for matching if their CGM data met sufficiency criteria. CD+ children were randomly matched with up to four CD−, using five clinical covariates to adjust for confounding from clinically-relevant physiologic states. Covariates were dichotomized to perform exact matching: sex, pubertal age (males ≥11.5 years [median age to attain testicular volume ≥4 mL [24]]; females ≥10 years [median age to observe Tanner stage II breast development in girls with BMI 10–84th percentile [25]]), longer diabetes duration (≥6 months), overweight (BMI z-score ≥1.0, corresponding to the overweight definition ≥85th percentile [5]) and above target hemoglobin HbA_1c_ ≥7.5 % (accepted target for children who do not utilize automated insulin delivery [5]). Subjects with identical combinations of matching covariates were combined into the same strata for more statistically efficient analyses. Combined race and ethnicity categories were recorded but not used for matching. Individuals with Hispanic race or ethnicity recorded were categorized as Hispanic and all other race/ethnicity categories represent non-Hispanic individuals.

### Prandial and summative glucose outcomes

Our prandial outcomes quantify prandial dynamics, identified as periods of transient glucose rise, peak (maximal excursion), fall and trough (post-prandial nadir) most likely to represent meals. The primary outcome was meal trough glucose, selected to operationalize the evaluation of post-prandial hypoglycemia. Secondary meal outcomes (Figure 1B) included starting glucose, peak glucose, meal glucose rise (peak minus start), rise velocity (rise divided by time from start to peak), meal glucose fall (absolute value of trough minus peak) and fall velocity (fall divided by time from peak to trough). Area under curve (AUC) was calculated from meal start to trough using the trapezoidal rule (Python sklearn.metrics.auc). All continuous meal outcomes were log-transformed due to skewness. Meal hypoglycemia was also assessed as a dichotomous outcome, defined as meal trough glucose <70 mg/dL. Meal outcomes were analyzed for all meals and then repeated analyzing a subset restricted to the first meal per day, to reduce meal-to-meal heterogeneity.

Summative CGM outcomes were mean glucose, glucose management indicator (GMI=3.31+[0.02392 × mean glucose]), standard deviation and percent time in the following ranges: 70–180 mg/dL (time in range), 70–140 mg/dL (time in tight range), >180 mg/dL, >250 mg/dL, <70 mg/dL and <54 mg/dL. Summative CGM outcomes were computed for the matched cohort using their raw, 30-day CGM data without interpolation or smoothing (as might be available in a clinical setting), via R package iGlu (4.0.1). These CGM metrics were analyzed for the full 24 h per day, then repeated limited to typical waking hours (6–12AM [26]), a proxy for the mealtime period.

### Meal annotation algorithm

Broadly, the meal annotation algorithm takes smoothed CGM data; identifies meals by searching for a trio of meal peak, trough, then preceding starting glucose; and extracts key CGM interstitial glucose (herein glucose) values. We extracted the CGM glucose values from the 30-day windows prior to each index date from the BCH Dexcom Clarity dataset (downloaded November 11, 2023) using R (version 3.6.2) on the BCH Enkefalos-v2 (E2) high-performance computing cluster (Linux operating system). Meal annotation was conducted using Python (version 3.9.13) with packages scipy (1.9.1), numpy (1.21.5), pandas (1.4.4) and sklearn (1.0.2).

First, for more reliable meal detection while minimizing non-physiologic fluctuations, missing glucose data were imputed using linear interpolation and high-frequency fluctuations >0.0005 Hz [27] were smoothed using a low pass filter. Then, peaks were identified during typical waking hours (6–12 AM) using scipy.signal.find_peaks to search the glucose data for features matching the following parameters: peak height ≥100 mg/dL, ≥2 h between peaks, rise ≥50 mg/dL above baseline and with elevated glucose ≥30 min duration. Similarly, troughs were identified as follows: trough glucose ≥15 min to ≤4 h after peak, fall ≥15 mg/dL below baseline for ≥15 min duration. Troughs were restricted to 6–3 AM to allow for a trough following a late-night meal while filtering out overnight fasting lows. The lowest trough glucose following each peak was selected. Last, the start of each meal was defined as the nearest glucose inflection from downtrending to uptrending glucose during waking hours ≤4 h before meal peak and with ≥50 mg/dL glucose increase from meal start to peak.

### Data sufficiency for inclusion

To be eligible for matching, individuals were required to have at least 14 days of CGM data with ≥0.5 qualifying meals per day on average. Meals were disqualified if glucose values (prior to imputation) were missing ≤15 min before or after meal start, peak or trough; or if, from meal start to trough, more than 30 % of expected glucose values were missing or the glucose rate of change was >6 mg/dL/min [28].

### Statistical analyses

Patient characteristics were compared between groups using Wilcoxon rank-sum or Fisher’s exact tests. Linear mixed models were used to compare meal outcomes between CD groups in the matched cohort with a fixed effect for matched strata (adjusting for confounding from covariates) and a random effect for subject (accounting for correlation between repeated measures from individuals). For the log-transformed outcomes, effect estimates represent the percent change in the CD+ relative to the CD− group, calculated by exponentiating the coefficient (beta), subtracting 1 and multiplying by 100. Mixed effects logistic regression was used to compare meal hypoglycemia between CD groups, using the same fixed and random effects. Rank-based van Elteren tests were used to compare summative CGM metrics between the CD groups, adjusting for matched strata. All prandial and summative CGM outcome calculations and statistical analyses were conducted using R (version 4.3.2) using two-sided 0.05 level tests.

### Data availability and reporting guidelines

The datasets and code generated and/or analyzed in this study are available from the corresponding author upon reasonable request. We used the STROBE reporting guideline [29] to draft this manuscript, and the STROBE reporting checklist [30] when editing.

## Results

### Study cohort characteristics

During the study period, 1,531 individuals were diagnosed with T1D and 921 had recorded CD serologies <1 year of diabetes diagnosis. Of these, 850 were age 2–21 years with recently recorded BMI and HbA_1c_ required for matching. Three hundred individuals also had CGM data available in the 30-day exposure window prior to CD serologies, and 288 met data sufficiency criteria, leaving 16 CD+ and 272 CD− individuals for matching.

The matched cohort comprised 16 CD+ and 60 CD− individuals: 13 CD+ matched exactly with four CD− individuals, two matched with three CD− individuals and one matched with two CD− individuals. The combined matched cohort was 70 % female and 75 % non-Hispanic White with median age 11.1 years (interquartile range 8.9–13.3), BMI z-score 0.23 (−0.15 to 0.61), diabetes duration 3.2 months (1.5–5.0) and HbA_1c_ 7.3 % (6.3–8.4). There were 2,067 days of CGM data analyzed (median 30 days/subject [25.8–30.0]) and 3,659 meals analyzed (median 48.5 meals/subject [34.5–62.5]). There were no statistically significant differences with respect to these characteristics between CD+ and CD− groups (Table 1).

**Table 1:** Matched cohort characteristics by celiac disease exposure status.

	CD− group	CD+ group
	n=60	n=16
Sex^a^, n, (% female)	42 (70)	11 (69)
Age^a^, years	11.6 (9.1–14.1)	10.6 (9.4–11.9)
BMI^a^ (z-score)	0.23 (−0.12–0.58)	0.36 (−0.07–0.78)
Diabetes duration^a^, months	3.1 (1.5–4.8)	4.3 (2.4–6.3)
Hemoglobin A_1c_^a^		
%	7.3 (6.3–8.3)	7.4 (5.8–8.9)
mmol/mol	56 (45–67)	57 (40–74)
Race/ethnicity, n (%)		
Asian	3 (5)	0
Black	3 (5)	1 (6)
Hispanic	3 (5)	0
White	45 (75)	12 (75)
Unknown^b^	6 (10)	3 (19)
Days of CGM data		
Total per group	1,628	439
Days per subject	30 (25.8–30.0)	30 (27.3–30.0)
Percent expected readings per subject	95 (91–99)	97 (94–99)
Meals analyzed		
Total per group	2,833	826
Meals per subject	47 (33–60)	57 (44–69)

Data are presented as median (interquartile range) unless otherwise indicated. There were no statistically significant differences between groups. CD−, no celiac disease; CD+, undiagnosed celiac disease; CGM, continuous glucose monitoring. ^a^CD+ matched to CD−on, dichotomized sex, age; BMI, diabetes duration and hemoglobin A_1c_. ^b^Unknown designates that the field was completed as “unknown”, “other”, or “declined to answer”.

### Prandial glucose dynamics

Our primary outcome, meal trough glucose, broadly operationalized the hypothesis that undiagnosed CD is associated with post-prandial hypoglycemia, evaluating for any change in trough glucose even if not meeting criteria for hypoglycemia. Using a linear mixed model, there was no significant difference in meal trough glucose between the as-yet undiagnosed CD+ group and the unexposed CD−group (−8.2 % effect estimate, 95 % CI, −20.1–5.5 %, p=0.23; Table 2). Undiagnosed CD was also not associated with meal hypoglycemia, defined as meal trough glucose <70 mg/dL (17.1 % CD+ vs. 13.9 % CD−meals, odds ratio 1.32, 95 % CI, 0.72 to 2.45, p=0.36).

**Table 2:** Prandial glycemic outcomes by celiac disease exposure status.

	CD− group	CD+ group	Effect estimate	p-Value
	n=60	n=16		
**All meals**				

Start, mg/dL	114 (96–137)	104 (94–130)	−5.8 % (−17.3–7.2 %)	0.37
Peak, mg/dL	213 (185–266)	186 (173–241)	−7.9 % (−16.6–1.8 %)	0.11
Trough, mg/dL	103 (85–134)	93 (83–117)	−8.2 % (−20.1–5.5 %)	0.23
Rise, mg/dL	90 (82–100)	80 (75–94)	−8.9 % (−14.9–−2.5 %)	0.009
Fall, mg/dL	100 (91–111)	91 (85–101)	−6.7 % (−13.3–0.5 %)	0.07
Rise velocity, mg/dL/min	1.24 (1.08–1.51)	1.21 (1.08–1.40)	−6.4 % (−14.4–2.2 %)	0.15
Fall velocity, mg/dL/min	0.79 (0.69–0.95)	0.84 (0.65–0.86)	−5.9 % (−13.6–2.5 %)	0.17
AUC, mg/dL/h	599 (490–684)	531 (436–646)	−8.8 % (−20.7–5.0 %)	0.20

**First meal per subject per day**				

Start, mg/dL	112 (96–136)	104 (96–132)	−3.7 % (−15.1–9.2 %)	0.56
Peak, mg/dL	214 (191–277)	196 (179–233)	−7.3 % (−16.2–2.4 %)	0.14
Trough, mg/dL	102 (81–131)	96 (83–119)	−5.3 % (−18.0–9.3 %)	0.46
Rise, mg/dL	105 (94–121)	96 (89–108)	−10.5 % (−17.8–−2.4 %)	0.01
Fall, mg/dL	91 (81–109)	83 (72–98)	−9.3 % (−16.–−1.5 %)	0.02
Rise velocity, mg/dL/min	1.35 (1.09–1.59)	1.31 (1.06–1.63)	−3.1 % (−12.3–7.0 %)	0.54
Fall velocity, mg/dL/min	0.79 (0.68–0.95)	0.76 (0.69–0.88)	−7.7 % (−15.3–0.7 %)	0.08
AUC, mg/dL/h	605 (513–860)	516 (454–646)	−9.6 % (−22.6–5.5 %)	0.20

Meal outcomes are presented as the group median (interquartile range) of the median outcome per individual. Outcomes were compared using linear mixed models adjusting for matched strata and accounting for correlation between repeated measures from individuals. Meal glucose rise was calculated as peak minus start glucose and rise velocity was calculated as rise divided by time from start to peak. Meal glucose fall was calculated as the absolute value of trough minus peak glucose and fall velocity was calculated as fall divided by time from peak to trough. All meal outcomes were log-transformed and effect estimates are presented as percent changes (95 % confidence interval). CD−, no celiac disease; CD+, undiagnosed celiac disease; AUC, area under curve.

**Figure 2:**
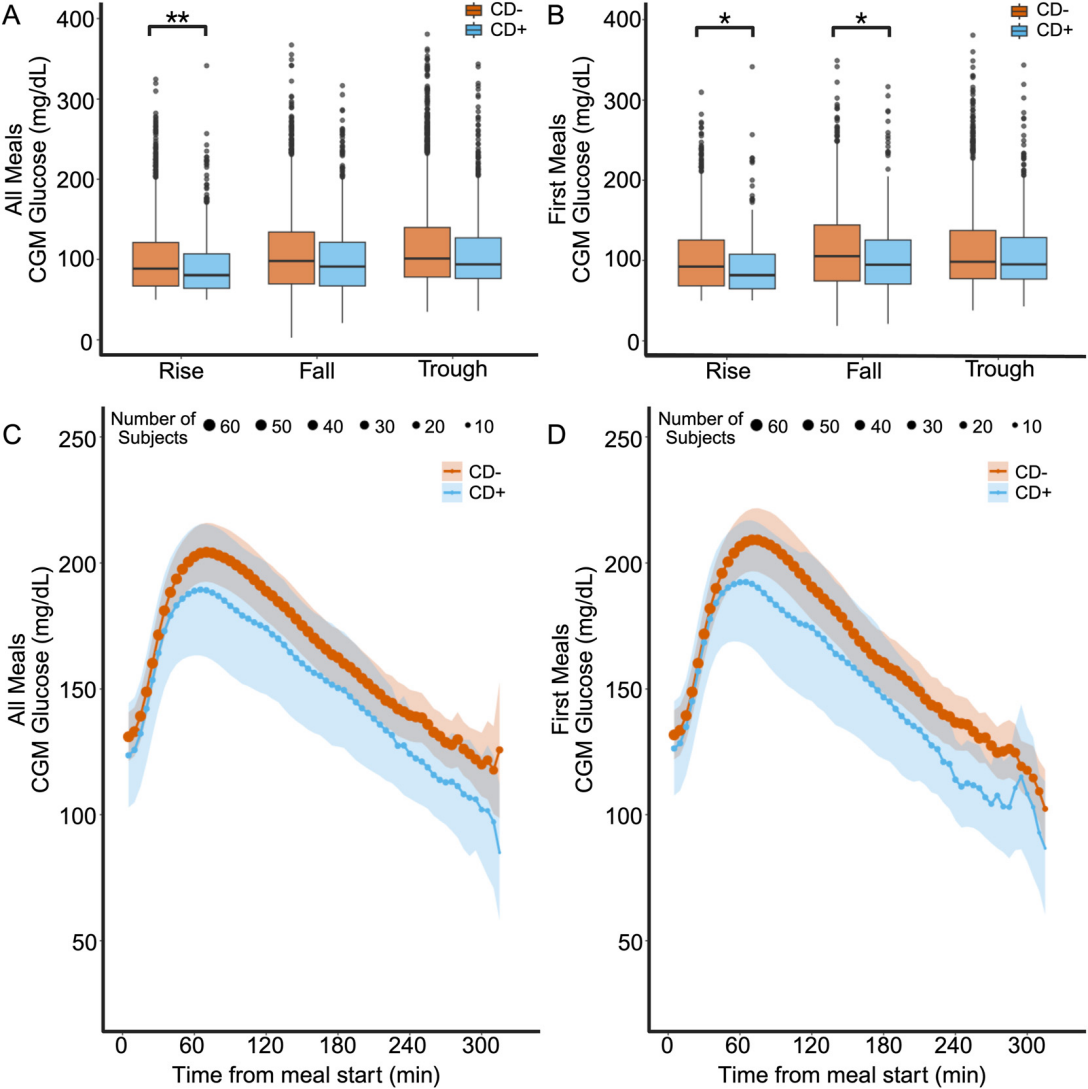
Blunted meal glucose excursions in youth with undiagnosed celiac disease and type 1 diabetes. Data from the CD−group are shown in orange and data from the CD+ group are shown in blue. (A–B) comparison of distribution of meal glucose rise, fall and trough for (A) all meals and (B) a subset consisting of the first meal per day (first meals). Rise was calculated as meal glucose peak minus start and fall was calculated as the absolute value of meal glucose trough minus peak. Boxes represent the interquartile range (IQR, 25th to 75th percentile). Horizontal lines inside the boxes denote median values, whiskers outside the boxes extend to 1.5 × IQR and dots denote outliers outside 1.5 × IQR. (C–D) mean of mean meal glucose per individual (solid circles) and 95 % confidence intervals (shaded regions) for (C) all meals and (D) the subset of first meals. Timepoint-wise mean glucose was calculated at each 5-min increment following meal start, first by individual, then across the CD groups. Meals were of variable durations and the number of individuals with data at each time increment is represented by circle size. To generate representative illustrations of meal glucose patterns, the longest 5 % of meals per individual were excluded, then the longest 5 % of all remaining meals were excluded. CGM, continuous glucose monitoring; CD−, no celiac disease; CD+, undiagnosed celiac disease; *p<0.05, **p<0.01. Created in BioRender. Ruiz, J. (2025) https://BioRender.com/ulrxr00.

We then assessed for other changes in prandial glucose dynamics in the setting of T1D and undiagnosed CD. The meal glucose rise (increase from start to peak) was significantly lower among CD+ compared to CD− (−8.9 % effect estimate, 95 % CI, −14.9–−2.5 %, p=0.009). The meal glucose fall (decrease from peak to trough) was also lower in the CD+ group, though not reaching statistical significance (−6.7 % effect estimate, 95 % CI, −13.3–0.5 %, p=0.07, Figure 2A). Similarly, other related meal outcomes including peak glucose, rise velocity, fall velocity and AUC had negative effect estimates but did not reach statistical significance (Table 2).

We then repeated the analysis with CGM meal data limited to the first meal of each day to reduce the impact of heterogeneity from inter-prandial glycemia and potential diurnal variation in insulin sensitivity and/or meal glycemic index. Within this subset of first meals, undiagnosed CD was again associated with a significantly reduced meal glucose rise (−10.5 % effect estimate, 95 % CI, −17.8–−2.4 %, p=0.01) and a significantly reduced meal glucose fall (−9.3 % effect estimate, 95 % CI, −16.5–−1.5 %, p=0.02, Figure 2B). The fall velocity was also slower in the CD+ group, though not reaching statistical significance (−7.7 % effect estimate, 95 % CI, −15.3–0.7 %, p=0.08). There were no significant differences between groups in trough glucose or the other secondary outcomes, though effect estimates were all negative (Table 2). Figure 2 illustrates representative CGM meal glucose data for all meals (Figure 2C) and the subset of first meals (Figure 2D).

### Summative glucose metrics

Last, we analyzed differences in 30-day summative CGM metrics (that might be available at a clinical visit) in relation to celiac status. Among individuals in the cohort, there was a median of 95.3 % (91.9–98.7 %) of expected CGM values available, with only one individual with <70 % of expected CGM values (56.6 %). The summative CGM metrics, including time below 70 mg/dL and time below 54 mg/dL, did not significantly differ between groups when calculated over the full 24 h per day or when limited to waking hours (Table 3).

**Table 3:** Summative continuous glucose monitoring metrics by celiac disease exposure status.

	CD− group	CD+ group	p-Value
	n=60	n=16	
**All data**			

GMI (%)	6.9 (6.5–7.7)	6.6 (6.3–7.5)	0.27
Mean, mg/dL	152 (132–183)	139 (126–174)	0.27
SD, mg/dL	48 (41–62)	44 (35–66)	0.32
Time in range, 70–180 mg/dL (%)	76 (53–85)	81 (60–89)	0.25
Time in tight range, 70–140 mg/dL (%)	48 (28–64)	57 (33–68)	0.27
Time above 180 mg/dL (%)	24 (14–46)	16 (9–40)	0.23
Time above 250 mg/dL (%)	4 (2–16)	2 (0–15)	0.13
Time below 70 mg/dL (%)	0.7 (0.3–1.6)	0.9 (0.3–2.0)	0.60
Time below 54 mg/dL (%)	0.07 (0.02–0.19)	0.06 (0.04–0.14)	0.95

**Waking hours (6 AM to 12 AM)**			

GMI (%)	7.0 (6.5–7.9)	6.7 (6.2–7.6)	0.17
Mean, mg/dL	155 (134–192)	141 (122–178)	0.17
SD, mg/dL	50 (42–63)	45 (35–66)	0.25
Time in range, 70–180 mg/dL (%)	72 (48–85)	81 (56–90)	0.17
Time in tight range, 70–140 mg/dL (%)	45 (25–61)	55 (31–71)	0.12
Time above 180 mg/dL (%)	27 (14–51)	17 (7–44)	0.13
Time above 250 mg/dL (%)	5 (1–19)	2 (0–14)	0.10
Time below 70 mg/dL (%)	0.8 (0.3–1.6)	1.0 (0.4–2.3)	0.44
Time below 54 mg/dL (%)	0.08 (0.01–0.21)	0.07 (0.05–0.18)	0.73

Data are presented as median (interquartile range). Metrics were compared between groups using van Elteren tests adjusting for matched strata. CGM, continuous glucose monitoring; CD−, no celiac disease; CD+, undiagnosed celiac disease; GMI, glucose management index; SD, standard deviation.

## Discussion

We demonstrate an attenuated meal glucose rise and fall in children with T1D immediately prior to CD diagnosis compared to matched individuals without CD, leveraging analyses of prandial CGM dynamics identified using a novel meal annotation algorithm. Importantly, our study showed there were no significant differences in prandial trough glucose, meal hypoglycemia or time hypoglycemic in this cohort of CGM users, despite the frequent citation of increased hypoglycemia as a clinical indicator of possible undiagnosed CD.

This is the first study to our knowledge that utilized CGM to assess the association between CD and glycemia just prior to CD diagnosis in a T1D cohort of CGM users, as prior CGM studies have focused on the impact of gluten-free diets after CD diagnosis [31], [32], [33]. Further, this study is the first within the dual T1D and CD population to apply an informatics methodology to retrospectively identify probable meal glucose excursions without meal diaries, in contrast to prior analyses requiring documentation of meal events [33]. While the algorithm was not tested on a dataset with verified meal events, it was developed using physiology-based parameters and systematically applied across the cohort. Our computational approach could aid researchers in automating assessment of prandial CGM events (e.g., meals) and dynamics (e.g., meal rise) within pre-existing CGM data without placing an additional burden of event logging on patients.

CD enteropathy causes damaged villi and dysmotility (delayed gastrointestinal transit [34]), both of which can drive prandial insulin-glucose mismatch via incomplete and delayed carbohydrate absorption. Thus, our findings of blunted prandial glucose rise, fall and slower fall velocity are concordant with the underlying pathophysiology of untreated CD. This was accentuated when restricting analyses to the first meal of each day (often containing more carbohydrates) and so CD malabsorption may be modulated by meal carbohydrate and/or gluten content. Despite fewer observations, the first-meal analyses strengthened several associations, likely by reducing heterogeneity from meal overlap, meal composition and diurnal changes in insulin sensitivity.

Complementary methods to identify patients at highest risk of CD are needed, as current guidelines lack quantitative glycemic measures of subclinical changes that indicate a need for CD testing. Pediatric guidelines recommend infrequent CD screening every 2–5 years [4], with less clear guidance beyond the first 5 years after T1D diagnosis [5], which can result in large gaps between screening. Our findings of attenuated prandial glucose rise and fall are novel, dynamic CGM biomarkers that, in future studies, could be refined, expanded upon and incorporated into a predictive model of CD in T1D. A predictive model can integrate multiple significant CGM and clinical features to help risk-stratify patients and guide CD evaluation, and could be integrated into CGM platforms, potentially enabling real-time celiac risk assessment without additional clinical burden.

Post-prandial hypoglycemia has been proposed as an identifiable glycemic feature to prompt testing for CD in T1D [4], 5]. However, in this cohort of CGM users, we observed no significant difference in post-prandial trough glucose, meal hypoglycemia or any summative CGM metrics standardly reviewed in a clinical encounter. Our results complement findings from prior studies of treated vs. untreated CD in T1D, in which CGM analyses revealed no difference in hypoglycemic episodes or other CGM metrics [31], 32]. There are several clinical factors that might explain why untreated CD was not associated with hypoglycemia in this cohort. Importantly, our cohort was exclusively comprised of individuals using CGM, which provides anticipatory alerts prompting intervention before hypoglycemia occurs. Providers may have lowered insulin doses for CD+ individuals experiencing hypoglycemia, reducing insulin-glucose mismatch. Further, this study assessed children during the first year following diabetes diagnosis (selected as a time of systematic CD screening in our practice), when some endogenous insulin and glucagon production remains and can mitigate hypoglycemia. Lastly, the degree of malabsorption and resulting glycemic sequelae in CD is variable and not reliably correlated with serologies. Nonetheless, these clinical factors and our findings argue against the primacy of unexplained hypoglycemia as the distinguishing glycemic feature of CD among children with T1D using CGM.

This retrospective study was strengthened by the matched cohort design, which adjusted for important physiologic covariates, including the increased risk of hypoglycemia in new-onset T1D as children enter remission and require less exogenous insulin. CGM data missingness was counterbalanced by numerous rules for data sufficiency. Additionally, CGM data assessment before CD serologies mitigated confounding from patient knowledge and potential gluten-free diet. Data were not uniformly available to adjust for additional effect modifiers, including insulin dosing, meal composition and endogenous β-cell function (e.g. c-peptide). HbA_1c_ was not uniformly measured at the index date, but the distribution of HbA_1c_ and GMI were similar among the cohort, indicating that HbA_1c_ measurements used were a reasonable approximation for glycemia during the exposure window. The cohort had a small sample size with limited racial/ethnic diversity and was restricted to children at a single academic medical center using CGM during the first year of diabetes, thus limiting generalizability beyond this context.

## Conclusions

In conclusion, this study leveraged a computational meal annotation algorithm to demonstrate blunting of meal glycemic excursions prior to CD diagnosis vs. no CD diagnosis in a matched cohort of children with new-onset T1D using CGM. Hypoglycemia did not differ between groups but is often cited as a glycemic feature of emerging CD in T1D. Thus, this work lays the foundation for a new risk-assessment paradigm leveraging prandial glycemic biomarkers beyond hypoglycemia to identify increased CD risk among CGM users. Findings from this and future work in broader T1D populations can inform a CGM-based model for real-time prediction of CD to help drive earlier diagnosis and mitigate the consequences of CD in T1D.
